# Optic nerve diameter in early-stage idiopathic Parkinson’s disease: A prospective observational study

**DOI:** 10.1097/MD.0000000000048676

**Published:** 2026-05-08

**Authors:** Osman Korucu, Arif Ülkü Yener, Selcan Ekicier Acar, Hatice Karaer Ünaldi, Volkan Savici, Ömer Akbudak, Burcu Karabulut, Sinem Fidan Buçin, Fatma Nur Birgin, Emine Emektar

**Affiliations:** a Department of Neurology, Atatürk Sanatorium Training and Research Hospital, Ankara, Turkey; b Department of Ophthalmology, Atatürk Sanatorium Training and Research Hospital, Ankara, Turkey; c Department of Emergency Medicine, Atatürk Sanatorium Training and Research Hospital, Ankara, Turkey.

**Keywords:** Idiopathic Parkinson’s disease, Optic nerve sheath diameter, Ultrasonography

## Abstract

Idiopathic Parkinson’s disease (IPD) is a neurodegenerative disorder affecting both motor and non-motor pathways, including the visual system. Although retinal changes have been proposed as biomarkers of neurodegeneration, data on optic nerve involvement in early IPD are limited. This study aimed to compare optic nerve sheath diameter (ONSD) between patients with early-stage IPD and healthy individuals. In this prospective observational study, 100 patients with IPD and 50 healthy controls were recruited between June 2021 and November 2025. All participants underwent comprehensive ophthalmologic examination and B-scan ultrasonography. ONSD was measured 3 mm posterior to the globe using a standardized technique. Patients with ocular, orbital, or systemic conditions affecting the optic nerve were excluded. Disease severity was assessed using the Unified Parkinson’s Disease Rating Scale Part III and the Hoehn–Yahr staging system. Statistical analyses were performed using non-parametric tests, with a significance level of *P* < .05. The median disease duration in the IPD group was 24 months, and most patients were classified as Hoehn–Yahr stage 1 or 2. There were no significant differences between patients with IPD and controls in right or left ONSD measurements. Subgroup analyses according to Hoehn–Yahr stage also showed no stage-dependent differences. Optic nerve sheath diameter appears preserved in patients with early-stage IPD and does not significantly differ from that of healthy controls. These findings suggest that ultrasonographic ONSD may have limited value as a structural biomarker in early disease stages. Further studies including advanced stages and multimodal imaging approaches are needed.

## 1. Introduction

Idiopathic Parkinson’s disease (IPD) is a progressive neurodegenerative disorder that affects more than 1% of individuals over the age of 65 years and is characterized by the gradual loss of dopaminergic neurons in the substantia nigra pars compacta. In recent years, accumulating evidence has demonstrated that Parkinson’s extends beyond its classical motor pathways and also involves the visual system, where dopamine plays a crucial neuromodulatory role. Retinal dopaminergic deficiency is associated with several non-motor visual disturbances, including impaired contrast sensitivity, reduced color discrimination, abnormalities in depth perception, and difficulty tracking moving objects.^[1,2]^

Advances in ocular imaging have further highlighted the fact that the retina undergoes measurable structural changes in the IPD. High-resolution optical coherence tomography studies conducted in the past few years have consistently shown thinning of the retinal nerve fiber layer, the ganglion cell complex, and the inner plexiform layer, supporting the concept that the retina mirrors central neurodegenerative processes.^[3,4]^ More recently, retinal alterations have been proposed as potential noninvasive biomarkers that can reflect disease severity and progression.^[5–7]^ These findings suggest that neurodegenerative changes in Parkinson’s may extend beyond the retina and involve the optic nerve. Although increased optic nerve sheath diameter (ONSD) is most strongly associated with elevated intracranial pressure, recent evidence indicates that ONSD enlargement is not entirely specific to intracranial hypertension. Since the optic nerve sheath is influenced by both cerebrospinal fluid dynamics and local orbital processes, several conditions, including optic neuritis, orbital inflammatory disorders, and papilledema of non-intracranial origin, may also lead to ONSD widening in the absence of true intracranial pressure elevation.^[8–10]^ However, despite the growing interest in ocular biomarkers, studies evaluating ONSD measurements in IPD are limited. This gap in the literature underscores the need to investigate whether optic nerve structural characteristics differ between individuals with IPD and healthy controls and whether these measurements may provide additional insight into visual pathway involvement in Parkinson’s disease.

The primary objective of this study was to evaluate optic nerve structural characteristics in patients with early-stage IPD and compare these parameters with those in healthy controls. A secondary objective was to investigate whether optic nerve measurements demonstrate any association with IPD disease severity.

We hypothesized that patients with early-stage IPD might demonstrate measurable differences in ONSD compared with healthy controls.

## 2. Materials and methods

This prospective observational study was conducted in the Neurology and Ophthalmology Department of Ankara Atatürk Sanatoryum Training and Research Hospital, a tertiary care center with a capacity of 780 beds that serves a large urban population. The hospital evaluates approximately 1000 patients with Parkinson’s disease annually. Ethical approval for the study was obtained from the Ankara Atatürk Sanatoryum Training and Research Hospital (Decision No: 2012-KEAK-15/2339; Date:22.06.2021). The study design adhered to the Strengthening the Reporting of Observational Studies in Epidemiology (STROBE) guidelines.

### 2.1. Study population

The study included patients aged 40 to 80 years with a confirmed diagnosis of IPD according to the Movement Disorder Society clinical diagnostic criteria. The study was conducted between June 15, 2021 and November 15, 2025.

The control group consisted of individuals without known ocular or neurodegenerative disease who presented to the neurology outpatient clinic for non-central neurological complaints, such as entrapment neuropathy, and met the inclusion criteria. All controls underwent neurological and ophthalmological evaluation to exclude conditions that could affect the optic nerve. Written informed consent was obtained from all participants before enrollment in the study. Patients with intracranial hypertension, pituitary tumors, Graves’ disease, a history of temporal arteritis or ischemic optic neuropathy, diabetes mellitus, previous globe trauma or vitreoretinal surgery, ocular/orbital tumors, Parkinsonism other than idiopathic Parkinson’s disease, or those diagnosed with secondary Parkinson’s disease were excluded from the study. The Unified Parkinson’s Disease Rating Scale (UPDRS) is a widely used clinical tool designed to evaluate the severity and progression of Parkinson’s disease in multiple functional domains. In our study, the UPDRS Part III (Motor Examination) was used to assess motor impairment. In addition, the Hoehn–Yahr scale was used for global staging of motor disability.

### 2.2. Ophthalmologic examination

Before the study, a fundus examination was performed to exclude any retinal pathology or optic nerve defects. Following this, the patient was seated, asked to close the eyes gently, and gaze downward. Retrobulbar optic nerve imaging was performed using a Quantel Medical Compact Touch/5952 (France) device equipped with a 15-MHz ultrasonographic B-scan probe.

ONSD was measured 3 mm posterior to the globe using a standardized technique. The device’s calibrated measurement tool was used to draw a transverse line from one automatically detected optic nerve border to the opposite border, after which the system calculated the diameter in millimeters. Measurements were performed in both eyes. All ultrasonographic evaluations, including ONSD measurements, were conducted by the same experienced clinician to ensure consistency. Inter- and intra-observer reliability analyses were not performed. However, all measurements were obtained by a single experienced operator using a standardized protocol, and this was considered to minimize measurement variability.

The sample size was calculated a priori for the comparison of the mean ONSD between the idiopathic Parkinson’s disease group and healthy controls. Based on previous studies reporting a standard deviation of approximately 0.3 to 0.4 mm for ultrasonographic ONSD measurements in healthy adults, we assumed a standard deviation of 0.40 mm and considered a between-group difference of 0.20 to 0.25 mm to be clinically relevant.^[11]^ Using a 2-sided independent-samples t-test with an alpha level of 0.05, a desired power of 80% (1–β = 0.80), and a group allocation ratio of 2:1 (PD/control), this corresponds to a detectable effect size of approximately Cohen’s d = 0.48 (moderate effect). Under these assumptions, a minimum of 96 patients with Parkinson’s and 48 controls were included. Our final sample of 100 patients with PD and 50 controls provided adequate statistical power for the primary comparison. All calculations were performed using the G*Power 3.1 software.

### 2.3. Statistical analysis

All data obtained during the study and recorded in the study forms were analyzed using the statistical software IBM SPSS 20.0 (Chicago). The distribution of continuous and discrete numerical variables was assessed using the Kolmogorov–Smirnov test to determine conformity with normal distribution. Continuous variables are presented as median (IQR 25 to 75), while categorical variables are expressed as the number of cases and percentages (%). Categorical variables were evaluated using the chi-square test and continuous variables were analyzed using the Mann–Whitney *U* test. To account for potential confounding effects of age and sex, an adjusted analysis was performed using a multivariable linear regression model, with ONSD as the dependent variable and group status, age, and sex as independent variables. Statistical significance was set at *P* < .05.

## 3. Results

A total of 100 patients with Parkinson’s and 50 healthy controls were included in this study. The median disease duration among patients with PD was 24 months (IQR, 9–48 months). Of these patients, 55% were classified as Hoehn–Yahr stage 1, 42% as stage 2, and 3% as stage 3.

Compared with the control group, Parkinson’s patients differed significantly in sex distribution (38% vs 56% female, *P* = .036) and age (median 70.5 vs 66.5 years, *P* = .022). However, there was no significant difference between the Parkinson and control groups regarding ONSD measurements (right ONSD: *P* = .330; left ONSD: *P* = .192). The results are presented in Table 1.

**Table 1:** Baseline characteristics and Optic nerve sheath diameter measurements in Parkinson’s disease and controls.

	Parkinson	Control group	*P* value
Woman gender, %	38 (38%)	28 (56%)	.036
Age (IQR 25–75)	70.5 (64.2–74)	66.5 (61.7–71)	.022
Right ONSD (IQR 25–75)	4.66 (4.28–4.99)	4.56 (4.31–4.83)	.330
Left ONSD (IQR 25–75)	4.73 (4.30–5.14)	4.52 (4.22–4.89)	.192

ONSD = optic nerve sheath diameter, Data are presented as median (IQR).

When patients were stratified according to Hoehn–Yahr staging, no significant differences in right or left ONSD values were observed between stage 1 and stage 2 Parkinson’s patients and their corresponding controls (*P* > .05). The comparisons are presented in Table 2.

**Table 2:** Optic nerve sheath diameter measurements in Stage 1–2 Parkinson’s disease versus controls.

	Stage 1 Parkinson	Control group	*P* value
Right ONSD	4.67 (4.27–5.00)	4.56 (4.31–4.83)	.347
Left ONSD	4.70 (4.21–5.10)	4.52 (4.22–4.89)	.399
	Stage 2 Parkinson	Control group	*P* value
Right ONSD	4.65 (4.29–4.95)	4.56 (4.31–4.83)	.485
Left ONSD	4.56 (4.31–4.83)	4.52 (4.22–4.89)	.156

ONSD: Optic nerve sheath diameter, Data are presented as median (IQR).

To account for the baseline differences in age and sex between the groups, a multivariable linear regression analysis was performed. After adjusting for these variables, the group status (IPD vs Control) was not independently associated with the mean optic nerve sheath diameter (B = −0.033, 95% CI: −0.226 to 0.159, *P* = .733).

## 4. Discussion

In this prospective observational study, we investigated ONSD in patients with early-stage IPD and compared them with those of a healthy control group. The findings demonstrated no significant differences in right or left ONSD measurements between patients with IPD and controls. Furthermore, subgroup analysis according to Hoehn–Yahr staging (stages 1 and 2) revealed no stage-dependent variation in ONSD values. These results suggest that optic nerve sheath diameter, as assessed using B-scan ultrasonography, appears structurally preserved in the early stages of idiopathic Parkinson’s disease. In addition, multivariable linear regression analysis adjusted for age and sex demonstrated that group status was not independently associated with optic nerve sheath diameter. This finding supports the primary unadjusted results and suggests that the absence of differences between groups was not explained by demographic imbalances.

The retina and optic nerve are embryologically derived from the central nervous system and exhibit structural and functional properties similar to those of the brain.^[12–14]^ Therefore, the retina is considered a potential biomarker for neurodegenerative brain diseases.

The optic nerve is composed of glial cells and axons of retinal ganglion cells, extending from the optic pathway to the lateral geniculate nucleus and superior colliculus, thus forming a direct connection with the brain.^[13]^

Several neurodegenerative disorders are characterized by α-synuclein (αSyn) aggregation and are classified as α-synucleinopathies, of which idiopathic Parkinson’s disease is the most common.^[15]^ Previous studies have reported that retinal αSyn pathology may correlate with disease severity, suggesting a possible role as a biomarker of neurodegeneration.^[16]^

Despite numerous studies examining α-synuclein accumulation in the retina, investigations focusing on the optic nerve are limited. A postmortem immunohistochemical study demonstrated that the detection of αSyn pathology in the retina and optic nerve showed high specificity (97%) and sensitivity (82%) for the presence of primary cerebral α-synucleinopathy. Notably, this study found that αSyn pathology in the optic nerve correlated more accurately with the cerebral αSyn burden than with retinal pathology alone. Lewy neurites in the retina and optic nerve as well as oligodendroglial cytoplasmic inclusions in the optic nerve have been identified.^[17]^ However, these findings are largely based on histopathological studies, and structural changes detectable by ultrasonographic measurements in the early stages of idiopathic Parkinson’s disease remain unclear.

Another study suggested that tau and α-synuclein may propagate along neural pathways, indicating that clinical assessment of visual function, particularly examination of the retina and optic nerve, could provide additional insights into disease progression.^[18]^ Determining whether pathological changes within the optic nerve are associated with measurable alterations in optic nerve diameter in idiopathic Parkinson’s disease may provide complementary information to other imaging-based biomarkers, such as neuromelanin imaging and quantitative susceptibility mapping, particularly in the early stages of the disease.^[19]^

However, in contrast to the clear retinal changes described in recent meta-analyses and systematic reviews, our study did not identify differences in optic nerve diameter between patients with IPD and controls. There are several possible explanations for this finding. First, neurodegenerative changes in IPD appear to be most pronounced at the level of retinal ganglion cells and inner retinal layers, which may undergo structural changes before alterations in the optic nerve trunk become detectable.^[15,20]^ Second, the optic nerve diameter measured by ultrasonography reflects a relatively coarse anatomical parameter; subtle axonal loss may not be sufficient to alter the total nerve–sheath complex. Third, the ultrasonographic optic nerve diameter is highly sensitive to changes in intracranial pressure, but it may lack sensitivity for chronic, slowly progressive neuroaxonal loss typical of IPD.

Recent reviews have also emphasized that not all ocular structures are equally affected in Parkinson’s disease. For example, some posterior segment structures, such as the choroid, appear relatively preserved despite thinning of the retinal layers.^[21]^ This suggests that structural involvement may not extend uniformly throughout the entire visual pathway. Consistent with this concept, our findings indicate that optic nerve sheath diameter does not appear to differ between patients with early-stage IPD and healthy controls, although retinal degeneration has been reported in previous studies.

This study has several strengths. First, it was designed as a prospective observational study with clearly defined inclusion and exclusion criteria. Second, all measurements were obtained using a standardized ultrasonographic technique. Third, potential confounding conditions affecting the optic nerve were carefully excluded. Finally, the sample size was determined using an a priori power calculation.

## 5. Limitations

This study has several limitations. First, the majority of patients were in Hoehn–Yahr stages 1 to 2; therefore, the findings may not be generalizable to patients with advanced disease. Second, the cross-sectional design did not permit the evaluation of longitudinal neurodegenerative changes. Third, intra- and inter-observer reliability analyses were not performed. Although all measurements were obtained by a single experienced examiner using a standardized protocol, ultrasonography remains an operator-dependent technique. Finally, the control group was recruited from patients presenting to the neurology outpatient clinic, which may have introduced a degree of selection bias.

## 6. Conclusion

In this prospective observational study, optic nerve sheath diameter did not differ significantly between patients with early-stage idiopathic Parkinson’s disease and healthy controls. These findings suggest that ultrasonographic optic nerve measurements may have limited value as a structural biomarker in the early stages of the disease. Further studies including patients with more advanced disease and incorporating multimodal imaging approaches are needed to clarify the potential role of optic nerve assessment in Parkinson’s disease.

## Acknowledgments

The authors have no acknowledgments to declare.

## Author contributions

**Conceptualization:** Osman Korucu, Selcan Ekicier Acar, Hatice Karaer Ünaldi, Burcu Karabulut, Sinem Fidan Buçin, Fatma Nur Birgin.

**Data curation:** Osman Korucu, Arif Ülkü Yener, Selcan Ekicier Acar, Hatice Karaer Ünaldi, Volkan Savici, Ömer Akbudak, Burcu Karabulut, Sinem Fidan Buçin, Fatma Nur Birgin, Emine Emektar.

**Formal analysis:** Osman Korucu, Arif Ülkü Yener, Selcan Ekicier Acar, Hatice Karaer Ünaldi, Ömer Akbudak, Burcu Karabulut, Sinem Fidan Buçin, Fatma Nur Birgin, Emine Emektar.

**Investigation:** Osman Korucu, Arif Ülkü Yener, Selcan Ekicier Acar, Hatice Karaer Ünaldi, Volkan Savici, Ömer Akbudak, Burcu Karabulut, Sinem Fidan Buçin, Fatma Nur Birgin.

**Methodology:** Osman Korucu, Arif Ülkü Yener, Selcan Ekicier Acar, Hatice Karaer Ünaldi, Volkan Savici, Ömer Akbudak, Burcu Karabulut, Sinem Fidan Buçin, Fatma Nur Birgin, Emine Emektar.

**Project administration:** Osman Korucu, Arif Ülkü Yener, Selcan Ekicier Acar, Hatice Karaer Ünaldi, Sinem Fidan Buçin, Fatma Nur Birgin, Emine Emektar.

**Resources:** Osman Korucu, Arif Ülkü Yener, Selcan Ekicier Acar, Hatice Karaer Ünaldi, Volkan Savici, Ömer Akbudak, Burcu Karabulut, Sinem Fidan Buçin, Fatma Nur Birgin, Emine Emektar.

**Software:** Osman Korucu, Arif Ülkü Yener, Hatice Karaer Ünaldi, Burcu Karabulut, Sinem Fidan Buçin, Emine Emektar.

**Supervision:** Selcan Ekicier Acar, Fatma Nur Birgin.

**Validation:** Osman Korucu, Arif Ülkü Yener, Selcan Ekicier Acar, Hatice Karaer Ünaldi, Volkan Savici, Ömer Akbudak, Burcu Karabulut, Sinem Fidan Buçin, Fatma Nur Birgin, Emine Emektar.

**Visualization:** Osman Korucu, Arif Ülkü Yener, Selcan Ekicier Acar, Hatice Karaer Ünaldi, Volkan Savici, Ömer Akbudak, Burcu Karabulut, Sinem Fidan Buçin, Fatma Nur Birgin, Emine Emektar.

**Writing – original draft:** Osman Korucu, Selcan Ekicier Acar, Emine Emektar.

**Writing – review & editing:** Osman Korucu, Hatice Karaer Ünaldi, Emine Emektar.
